# Challenging the Kauzmann paradox using an ultra-stable perfluoropolymer glass with a fictive temperature below the dynamic VFT temperature

**DOI:** 10.1038/s41598-023-31074-0

**Published:** 2023-03-14

**Authors:** Amer A. El Banna, Gregory B. McKenna

**Affiliations:** 1 Texas Tech University, Lubbock, TX USA; 2 North Carolina State University, Raleigh, NC USA

**Keywords:** Materials science, Soft materials, Glasses, Engineering, Chemical engineering

## Abstract

Ultra-stable fluoropolymer glasses were created using vacuum pyrolysis deposition that show large fictive temperature T_f_ reductions relative to the glass transition temperature T_g_ of the rejuvenated material. T_f_ was also found to be 11.4 K below the dynamic VFT temperature T_VFT_. Glass films with various thickness (200–1150 nm) were deposited onto different temperature substrates. Glassy films were characterized using rapid-chip calorimetry, Fourier-transform infrared spectroscopy and intrinsic viscosity measurements. Large enthalpy overshoots were observed upon heating and a T_f_ reduction of 62.6 K relative to the T_g_ of 348 K was observed. This reduction exceeds values reported for a 20-million-year-old amber and another amorphous fluoropolymer and is below the putative Kauzmann temperature T_K_ for the material as related to T_VFT_. These results challenge the importance of the Kauzmann paradox in glass-formation and illustrates a powerful method for the exploration of material dynamics deep in the glassy state (T_f_ < T < T_g_).

## Introduction

Thin films have become a vital component in our daily lives, whether it be in microelectronics, auto-mobiles, household appliances or food packaging. Thin film properties can differ drastically from bulk material properties, and this has resulted in considerable interest in the science and engineering communities. Thin film applications depend on the properties of the film and these depend on the fabrication technique. The effects of an enhanced mobility surface layer on such films has an increasing effect on the film properties with decreasing film thickness. Some of these effects include T_g_ depression and enhanced optical properties. Not only do thin films allow for easier integration into different devices, but they also allow fabrication to be tunable to the desired application requirements as these important film properties change with decreasing thickness, generally at sizes below 100 nm^1–14^.

While spin coating is a common method of making ultrathin polymer films, in the present work we focus on vapor deposition of polymers. This approach differs somewhat from the physical vapor deposition of small molecule organics, but builds on those ideas as a means of creating very stable glassy states. Furthermore, the interest in thin glassy polymer films has increased due to their enhanced properties that include wear resistance, thermal stability and tunable optical properties^15^.

Glasses are non-equilibrium materials that have a molecular structure that is kinetically trapped and that, consequently, shows a constant evolution towards equilibrium that is determined by both absolute temperature and the history of the glass formation and use conditions^16,17^. The glass transition temperature (T_g_) is the temperature at which the molecular mobility becomes slow relative to the cooling rate and the thermodynamic state-like variables (e.g., volume and enthalpy) begin to depart from the equilibrium state. Once below T_g_, the molecules evolve towards equilibrium through a process called structural recovery, also termed as aging. The fictive temperature (T_f_) is similar to T_g_ but is prominent for materials that have undergone structural recovery and is used as a measure of the non-equilibrium state that reflects the 'frozen-in' liquid structure of the glass. T_f_ is used to describe the material T_g_ for that structure, and the limiting T_f_ (T_f_′) is the T_g_ of the material measured after cooling by a heating experiment at the same rate. The T_f_ evolution with cooling rate or with isothermal aging is a measure of a material’s structural recovery towards equilibrium; the greater the reduction in T_f_ during an aging or slow cooling treatment, the more thermally stable the material is^17–23^.

Earlier work from the McKenna laboratory on a 20-million-year-old amber has shown that aging of the amber for this time scale resulted in a T_f_ reduction of 43.6 K relative to the T_g_ = T_f_′ and this accompanied a densification of the ancient amber of 2.1% relative to the density of the thermally rejuvenated material^23^. Experiments on the dynamics in the temperature window between the reduced fictive temperature and the T_g_ also showed a breakdown in the Vogel^24^-Fulcher^25^-Tammann^26^ (VFT) relationship which is associated with apparently diverging timescales at the notional thermodynamic temperature (Kauzmann^27^ temperature, T_K_) above 0 K, and which developed from the observation that the entropy of the supercooled liquid extrapolates to values lower than that of the crystal. This deviation of the dynamics from the VFT extrapolation is consistent with literature data obtained from long time aging experiments^17–23,28–30^ nearer to the glass transition temperature.

Another way to obtain low fictive temperature glasses has recently become of interest, that of physical vapor deposition. For example, Ediger and coworkers^31–39^ were able to “hike down the energy landscape” using physical vapor deposition (PVD) methods to produce stable films of small molecule glass formers grown at different deposition temperatures. Postulated to be due to an enhanced mobility of the depositing material, Ediger and coworkers’ results identified an optimum substrate temperature for deposition to be in the vicinity of 0.85T_g_, T_g_ being in K and is the nominal glass transition temperature generally obtained at a cooling rate of 10 K/min. Although Boucher et al.^40^ were successful in creating low fictive temperature polystyrene films by aging, the time associated with conducting such experiments is considerably longer than VPD experiments (orders of magnitude). In addition, those results did not account for the possibility that the extremely thin films have a lower T_K_ than the bulk material as is suggested by dynamical measurements in the thin film and nanoconfined polymer literature^1,11–14^.

Swallen et al.^32^ conducted PVD experiments with 1,3,5-(tris)napthylbenzene (TNB) and indomethacin (IMC) and reported T_f_ reductions of 33 K and 29 K, respectively. León-Gutierrez et al.^41–44^ worked on creating stable glasses of toluene and ethylbenzene and observed T_f_ reductions as well as a shift to the onset or devitrification temperature to higher temperatures, indicating an increase in glassy stability. Raegen et al.^45^ deposited poly (oligomeric) styrene and report an approximately 25 K T_f_ reduction as well as densification as large as 1.6%.

Bowie and Zhao^46^ simulated linear polymer thin film growth under vapor deposition polymerization where they varied the ratio, G, of the diffusion coefficient to the deposition rate and very high G values yielded high density polymer films. Lin et al.^47^ used molecular dynamics simulations to investigate properties of vapor deposited glasses created from short polymer chains and found that the deposition rate plays an important role in the stability of the deposited polymer glass and the deposited glass experienced higher density as well as higher kinetic stability. Additional work from Samanta et al.^48^ explored the differences in stability of vapor deposited organic glasses, 1,3-bis(1-naphthyl)-5-(2-naphthyl)benzene (TNB) and 9-(3,5-di(naphthalen-1-yl)phenyl)anthracene (α,α-A), with their liquid quenched counterparts, observing a density increase as well as improved kinetic stability for the VPD glasses. Dalal et al.^49^ used spectroscopic ellipsometry to better understand the properties vapor deposited ααβ-TNB glasses and found that the vapor deposited glass was denser as well as had a higher onset temperature for devitrification.

The above works have been generally interpreted to imply that the freedom allowed by VPD for the deposited molecules to explore a lower potential energy on the substrate’s surface allows packing densities to be obtained that would require orders of magnitude less time than that associated with physical aging experiments, yet leads to a glass that can be considered to have the same physical properties as a very long-time aged material. This interpretation, while acceptable, remains to be fully validated.

In recent work^50^ from the McKenna laboratory, vapor deposition experiments were performed using a high molecular weight (MW) amorphous fluoropolymer (Teflon AF 1600, MW = 400 kg/mol) and the results showed very large enthalpy overshoots corresponding to T_f_ reductions of up to 57 K, just above the reported T_K_ (the associated T_VFT_) by 0.2 K. The idea behind the vapor deposition and formation of high MW stable polymer films was that the polymer chains pyrolyze and repolymerize on the substrate temperature, resulting in stable glasses that have lower MW than the virgin material, but also remain polymeric, thus improving mechanical toughness needed for viscoelastic testing. They^50^ referred to this as vacuum pyrolysis deposition (VPD).

In the current work, we used vacuum pyrolysis deposition (VPD) to create highly stable glasses from a different perfuorinated polymer (CYTOP, fluoropolymer). The potential advantage of this material over the AF 1600 is that the glass transition temperature of the CYTOP is over 50 K lower than that of the AF 1600, which made viscoelastic testing in the atomic force microscope potentially easier. The stable glasses were characterized using rapid-chip calorimetry, Fourier transform infrared spectroscopy (FTIR) and intrinsic viscosity measurements. These latter measurements provided the picture of the VPD methodology leading to decreased molecular weight of the polymer, but that the thin film remained polymeric and had the same chemical structure as the original Teflon AF 1600. We compare results with those for the virgin CYTOP material using the same techniques, except for the calorimetry, in which case conventional DSC was also employed for some of the calorimetric measurements. We describe how the stable glasses were created and how we characterized their stability, as well as provide evidence that the material is still polymeric in nature.

## Experimental section

### Sample preparation

CYTOP (Type S) was purchased directly from Japan through AGC inc. (formerly Asahi Glass Co.). The reported ${M}_{W}$ from the manufacturer is 250,000–300,000 g/mol with a reported T_g_ of 108 $^\circ$C (381.2 K). The CYTOP was received in a solution of 9% CYTOP on weight basis. The solvent used for the solution was CT-Solv-180 (perfluorotributylamine). To get CYTOP in solid form, a known quantity of the solution was placed in a Pyrex container and subjected to a series of vacuum oven drying steps at different temperatures: 80 $^\circ$C × 24 h + 120 $^\circ$C × 12 h to remove dissolved air, 180 $^\circ$C × 24 h to evaporate the solvent and 240$^\circ$C × 24 h to remove bubbles by annealing. The drying process was considered complete once there was no decrease in weight after annealing at 240$^\circ$C. Once the sample was dried, it was allowed time to cool and the bulk CYTOP was removed from the mold. VPD was used to create ultra-stable CYTOP films with thickness ranging from 180 to 1150 nm. The VPD process was achieved using a Varian high vacuum evaporator (Varian 3118) and the deposition rate was tracked and confirmed using a quartz crystal microbalance (QCM). A temperature-controlled surface was used upon which the substrates were attached. Silicon wafers, mica sheets and Flash-DSC (FDSC) chips were used as the substrate at which the films were grown. Due to the design of the FDSC chips, film growth occurs on the back side of the chip with a mask used to ensure that the deposition area is limited only to the chip’s sensor area. The temperature of the substrate surface was varied between 0.79 and 0.91 T_g_, where T_g_ is in K and is the T_g_ measured in our labs of 104 °C (377.2 K) for the bulk material. We remark that the deposition temperatures were originally chosen based upon the manufacturer’s reported T_g_ = 108 °C (381.2 K), but we report our results based on our measured value. The deposition rate was maintained at approximately 0.1 nm/s by adjusting the current to the basket in the vacuum jar but basket temperature was not measured. The vacuum pressure achieved was in the range of 10^–7^ Torr^50^.

Bulk CYTOP samples (oven-dried) used in FTIR measurements were created using a mica sheet to spread a drop of CYTOP solution on the surface of a CaF_2_ IR window followed by oven drying. The VPD samples produced for FTIR analysis were deposited directly on NaCl and CaF_2_ windows. Handling of these windows with deposited samples was done with great care and after deposition the windows were placed in a desiccator to minimize exposure to humidity.

The as-received CYTOP solution was diluted to make solutions of the bulk polymer for intrinsic viscometry measurements. Solution concentrations varied between 0.01 and 0.18 mg/mL. For for the VPD samples, 1.1 µm thick films, grown on mica sheets, were placed directly in the solvent for 24 h to allow for the sample to dissolve into the solvent, yielding concentrations varying from 0.05 to 0.18 mg/mL.

### Calorimetric measurements

The fictive temperatures of the ultra-stable CYTOP were measured using a Mettler Toledo Flash Differential Scanning Calorimetry (FDSC) with Freon intercooler and nitrogen purge. Relevant temperature scans were conducted at a cooling and heating rate of 600 K/s. The heating scans were run in the temperature range of − 10–210 $^\circ$C, where the sample was held for 5 s and cooled back down to − 10$^\circ$C. The approximate sample mass of 180–220 nm thick CYTOP films ($\rho$ = 2.03 g/cm^3^) ranged between 91 and 112 ng. Information regarding the calibration of the Flash DSC is mentioned elsewhere^51^. For the ultra-stable CYTOP samples, 2 rejuvenated scans were conducted to ensure stability and reproducibility. The virgin material experiments were conducted using a Mettler Toledo DSC 822e. In this case the heating scans were conducted at 10 K/min on a 40 $\mu$L aluminum pan containing 15.76 mg of virgin CYTOP.

### FTIR measurements

FTIR measurements were performed on the VPD CYTOP as well as bulk CYTOP, both at different thicknesses, using a Bruker Optics VERTEX 70 Fourier transform infrared (FTIR) spectroscope. Each spectrograph is an average of 64 scans with a resolution of 4 cm^−1^.

### Intrinsic viscosity measurements

Intrinsic viscosity measurements were conducted to estimate the molecular weight of the VPD CYTOP. Bulk CYTOP solution was diluted to the desired concentration levels and used to determine the relative (${\eta }_{r}$) and specific (${\eta }_{sp}$) viscosities. A micro-Ostwald viscometer (2 mL) was used in a water bath with constant temperature of 25 °C. Each concentration viscosity was an average of three to five measurements. The average efflux time (${t}_{0}$) for pure solvent CT-Solv-180 was 1395 $\pm$ 4 s.

## Analysis

### Calorimetry

The fictive temperature, ${T}_{f}$, is the temperature at which the extrapolated equilibrium liquid line intersects the glass line on an enthalpy-temperature or volume-temperature plot. In calorimetry studies, ${T}_{f}$ is measured from a heating heat flow scan after cooling and is a function of the cooling rate. As the cooling rate decreases, ${T}_{f}$ also decreases as more time is allowed for the molecules to maintain equilibrium. ${T}_{f}$ is also a function of aging time and at long aging times, ${T}_{f}$ approaches the aging temperature ${T}_{a}$. Determination of ${T}_{f}$ was carried out using Moynihan’s method^51,52^ (Eq. 1) of area matching:

1 $$\int_{{T_{f} }}^{{T \gg T_{g} }} {\left( {C_{pl} \left( T \right) - C_{pg} \left( T \right)} \right)dT} = \int_{{T \ll T_{g} }}^{{T \gg T_{g} }} {\left( {C_{p} \left( T \right) - C_{pg} \left( T \right)} \right)dT}$$

here ${C}_{pl}$ and ${C}_{pg}$ are the liquid and glass heat capacities and ${C}_{p}$ is the apparent heat capacity of the material, in this case CYTOP.

Upon integration of Eq. (1) and applying it for 2 scans, the first (ultra-stable response) and second (rejuvenated response) heating scans, we reach to Eq. (2) from which the ${T}_{f}$ reduction is calculated:

2 $$\Delta T_{f} = T_{f,rejuv.} - T_{f,VPD} = \frac{{ - \left[ {H_{rejuv.} - H_{VPD} } \right]}}{{\Delta C_{p} }}$$

here ${T}_{f, rejuv.}$ and ${T}_{f, VPD}$ are the fictive temperatures of the rejuvenated and stable materials, ${H}_{rejuv.}$ and ${H}_{VPD}$ are the enthalpies obtained from the heating scans for the rejuvenated and stable materials and $\Delta {C}_{p}$ is the difference between heat capacities of the liquid and glass lines at ${T}_{f}$.

The cooling rate dependence of ${T}_{f}$ was calculated using a variation^51,52^ of the Vogel-Fulcher-Tammann (VFT) Equation^24–26^ that is expressed in terms of cooling rate, as shown in Eq. (3):

3 $$q = Aexp\left( {\frac{ - B}{{\left( {T_{f} - T_{0} } \right)}}} \right)$$

here $q$ is the cooling rate, $A$ and $B$ are fitting parameters and ${T}_{0}$ is the temperature at which the dynamics diverge to infinity.

Using the VFT parameters, the fragility ($m$) and the activation energy (${E}_{g}$) were calculated using Eqs. (4), (5)^51–54^:

4 $$m = \left( {\frac{{d\left[ {\log \left( q \right)} \right]}}{{d\left( {\frac{{T_{g} }}{T}} \right)}}} \right)_{{T = T_{g} }} = \frac{{B/T_{g} }}{{2.303\left( {1 - \left( {\frac{{T_{0} }}{{T_{g} }}} \right)} \right)^{2} }}$$

5 $$E_{g} = \frac{RB}{{\left( {1 - \left( {\frac{{T_{0} }}{{T_{g} }}} \right)} \right)^{2} }}$$

The Mauro-Yue-Ellison-Gupta-Allan (MYEGA)^55^ viscosity model was also used for comparison, expressed in terms of cooling rate:

6 $$\log_{10} q\left( T \right) = \log_{10} q_{\infty } + \frac{K}{T}exp\left( \frac{C}{T} \right)$$

here K and C are fitting parameters.

### Intrinsic viscosity measurements

The relative (${\eta }_{r}$) and specific (${\eta }_{sp}$) viscosities were determined using Eqs. (6), (7) shown below:

7 $$\eta_{r} \approx \frac{\eta }{{\eta_{0} }} \approx \frac{t}{{t_{0} }}$$

8 $$\eta_{sp} = \eta_{r} - 1 \approx \frac{{\eta - \eta_{0} }}{{\eta_{0} }} \approx \frac{{t - t_{0} }}{{t_{0} }}$$

here ${t}_{0}$ is the solvent’s measured efflux time and $t$ is the efflux time for the dilute polymer solution.

The intrinsic viscosity ($\left[\eta \right]$) is obtained by extrapolating to 0 polymer concentration the data resulting from normalizing $\mathrm{ln}{\eta }_{r}$ and ${\eta }_{sp}$ with concentration ($C$) as shown in Eq. (7).

9 $$\left[ \eta \right] = \mathop {\lim }\limits_{C \to 0} \left( {\frac{{\eta_{sp} }}{C}} \right) = \mathop {\lim }\limits_{C \to 0} \left( {\frac{{\ln \eta_{r} }}{C}} \right)$$

The Mark-Houwink Staudinger-Sakurda^56^ relation allows us to estimate the viscosity average molecular weight using the intrinsic viscosity as shown Eq. (8):

10 $$\left[ \eta \right] = K\left( {\overline{{M_{v} }} } \right)^{\alpha }$$

here $K$ is the Mark-Houwink parameter and $\alpha$ is the exponent, typically ranging from 0.6 to 0.8 for most polymers.

Applying Eq. (8) to both the VPD and virgin materials and subtracting them from each other results in Eq. (9) from which the VPD material’s viscosity average molecular weight could be estimated:

11 $$\overline{{M_{v, VPD} }} = \overline{{M_{v, VM} }} exp\left( {\frac{1}{\alpha }\ln \left( {\frac{{\left[ \eta \right]_{VPD} }}{{\left[ \eta \right]_{VM} }}} \right)} \right)$$

here $\overline{{M }_{v, VPD}}$ and ${M}_{v, VM}$ are the viscosity average molecular weight of the VPD and virgin materials.

## Results and discussion

### Fourier-transform infrared spectroscopy (FTIR)

**Figure 1:**
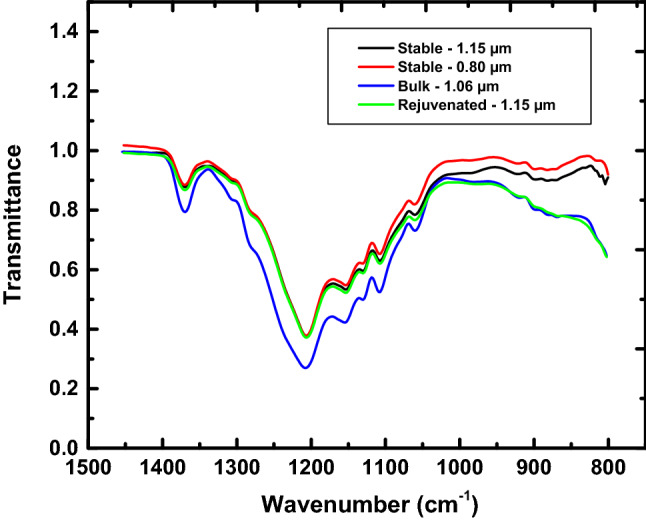
FTIR spectrographs for stable and bulk (virgin) CYTOP films at various thickness, as indicated. Peaks in the range of 1100–1200 cm^−1^ are due to –CF_2_ and peaks in the range of 1340 cm^−1^ are associated with -CF.

VPD was used to create stable glasses of amorphous fluoropolymer CYTOP. Due to the pyrolysis process, it is of interest to ensure that the stable glass formed has the same chemical structure as the virgin material (Though, importantly, for the purposes of the present ultra-stable glass investigation, this is a side issue as the physics of the VPD ultra-stable glass are compared with those of the same material but after thermal rejuvenation, i.e., after heating above T_g_). FTIR was utilized to explore the structure of the stable glasses formed. Figure 1 show the IR spectrograms for bulk and stable CYTOP films of various thickness. The main peaks shown in Fig. 1 are that of CF_2_ (symmetric at 1100 cm^-1^ and asymmetric at 1200 cm^−1^)^57^ and a CF stretch mode at 1340 cm^-1^. High thicknesses resulted in transmittance saturation in the spectral regions of interest and fabrication of a thinner sample (1.06 m$\mu$) was necessary to be able to compare the very thin VPD CYTOP samples to the virgin material. The spectrographs for the stable and rejuvenated CYTOP are in good agreement, while that of the bulk CYTOP agrees with peak position but differs slightly in intensity. Importantly, after rejuvenation, the VPD CYTOP film’s spectrograph agreed well with the bulk CYTOP film. The results shown in Fig. 1 show that the chemical structures of the VPD material and the as-received material are similar, concluding that the VPD of CYTOP didn’t produce a material of a different chemical structure. Similar results have also been reported for studies on stable and bulk fluoropolymers^50,58^.

The FTIR and intrinsic viscosity studies were carried out to provide information about the present polymer. While we could not determine the molecular weight distribution we could determine/estimate the intrinsic viscosity based molecular weight and the major result for these parts of the present investigation is that the CYTOP seems to retain its chemical structure (FTIR) and it remains polymeric, though with a lower molecular weight than the virgin material. Since perfluoropolymers are highly stable, once the vapor deposited material has formed, there is no evidence that the thermal exposures during subsequent testing would chemically change the material response. Nason et al.^59^ created amorphous fluoropolymer films using VPD and found little change in overall composition when compared to the virgin material. The results led to the conclusion that the volatile fragments resulting from cleavage takes place between two dioxole fragments (there are more of these junctions per chain than any other type), due to the high steric congestion at those points, and the free radical created can be stabilized by a neighboring O atom. Blanchet^58^ caried out laser ablation experiments on amorphous fluoropolymers in which the laser-induced pyrolytic decomposition yielded monomers that ejected at high velocities that impinge on the substrate and repolymerize to form a film. Regardless, the important aspect of the present work is that the vapor deposited, stable film is chemically the same as its rejuvenated counterpart. Thus, differences in physical response between the VPD films and their rejuvenated references are relevant to the comparisons to be made in the present work. Furthermore, fluoropolymers are quite stable in general, thus the mild heating treatments for the calorimetric measurements are not likely to lead to chemical degradation. Furthermore, the fact that the thermograms of the samples overlap in the liquid states after multiple heating cycles is consistent with there being no mass loss during the experiments.

### Intrinsic viscosity measurements

**Figure 2:**
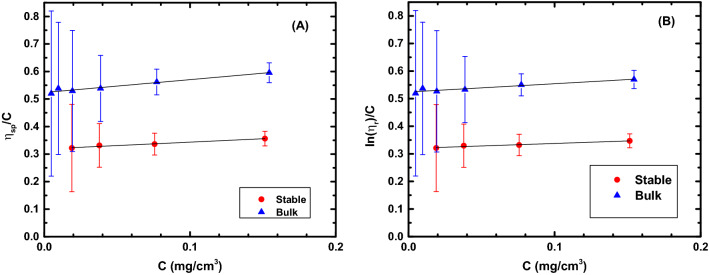
Intrinsic viscosity measurement results for VPD and bulk CYTOP. (**A**) Ratio of the specific viscosity (${\eta }_{sp}$) to concentration plotted versus concentration, with the intercept being the intrinsic viscosity. (**B**) Ratio of the natural logarithm of the relative viscosity (${\eta }_{r}$) to concentration plotted against concentration, with the intercept being the intrinsic viscosity. Best fit lines also shown for each data set.

Intrinsic viscosity measurements were conducted to determine the molecular weight of the stable CYTOP glasses created using VPD. Since in the prior work it was found that the chains of the AF 1600 amorphous Teflon pyrolyze and re-polymerize on the deposition substrate. Calculating the MW allows us to determine whether if the material is still polymeric in nature. Capillary viscometry measurements were made on different concentration solutions of both the untreated CYTOP and the VPD stable CYTOP materials and the relative and specific viscosities were calculated, from which the intrinsic viscosities were extracted. Intrinsic viscometry results show that $\left[\eta \right]$ dropped by approximately 38%, from 0.52 $\pm$ 0.01 to 0.32 $\pm$ 0.01 dL/g. Rearrangement of the Mark-Houwink-Sakurada relation, Eq. (8), which relates the intrinsic viscosity to the viscosity average MW, $\overline{{M }_{v}}$, results in a $\overline{{M }_{v}}$ in the range of 100–163 kg/mol, which indicates that the material is still polymeric in nature. Figure 2 below summarizes the intrinsic viscosity measurement results.

An analysis to determine if the cause of the molecular weight decrease was due to branching of the polymer chains during re-polymerization was carried out and is presented in Section 1 of the Supplemental Information 1. The analysis illustrates that the apparent MW decrease was not due to branching.

### Calorimetry

**Figure 3:**
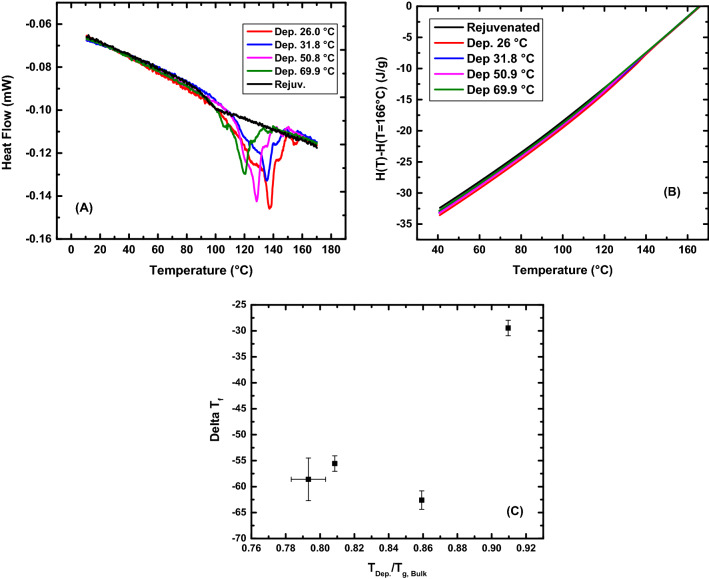
Summary of the VPD CYTOP calorimetry results. (**A**) Heat flow curves for stable CYTOP films of similar thickness (185–230 nm) deposited at different substrate temperatures. (**B**) Enthalpy curves for the stable CYTOP films created compared to the rejuvenated material. Enthalpy curves were calculated by integrating the heat flow curves. (**C**) T_f_ reduction (delta T_f_) relative to the rejuvenated CYTOP as a function of the ratio of deposition temperature and T_g, Bulk_. T_dep_ was determined based on reported T_g_ not the measured T_g_.

The major interest of the VPD method is the creation of glasses that are stable in the sense of having a reduced fictive temperature relative to that of the rejuvenated counterpart. It then is important to be able to characterize the stability of the glasses created and the determination of the T_f_ was the route chosen. Fast-scanning differential scanning calorimetry (FDSC) as well as regular differential scanning calorimetry (DSC) were used to characterize the stability of the stable CYTOP films as well as the virgin material, respectively. The stable glasses were grown by vapor deposition on substrates having different temperatures in order to explore the possibility of an optimum deposition T_Dep_/T_g,Bulk_ ratio. Figure 3A shows the heat flow curves for the stable glasses deposited at different deposition temperatures along with the rejuvenated material’s heating curve. Figure 3B shows the enthalpy evolution for the respective heat flow scans, the product of integrating the heat flow scans. Figure 3C shows the reduction in T_f_ for the stable material when compared to the rejuvenated sample’s T_f_, measured at the nominal heating rate of 10 K/min, after cooling at 10 K/min, being 75.2 $\pm$ 1.1 °C (348.3 K).

A summary of the results obtained through the calorimetry measurements is shown in Table 1. The largest T_f_ reduction was observed at a T_dep_/T_g,Bulk_ ratio of 0.86, which is consistent with literature reports. T_f_ reductions at lower ratios were also very large as evident from their undershoots in Fig. 3A. The highest T_dep_/T_g,Bulk_ ratio showed the smallest reduction in T_f_, which is also consistent with literature reports. Of importance to mention, the rejuvenated T_f_ for the stable film grown at 50.8 °C has been adjusted by + 3 °C. We associate the observed difference in the rejuvenated T_f_ as being due to variability in the chip sensors used for the vapor deposition. For example, Koh et al.^60^ performed structural recovery experiments on a single polystyrene thin film using nano-calorimetry and out of the four chips used in the study, differences of up to 4.46 K were observed from the instrument reported temperature.

**Table 1:** Fictive temperatures and fictive temperature reductions for stable CYTOP films deposited at different substrate temperatures. All Cooling rates were 0.167 K/s (10 K/min) and all heating rates were 600 K/s. $\Delta$ T_f_ value is relative to the rejuvenated T_f_.

T_dep_ (°C)	T_dep_/T_g,Bulk_	Rejuvenated T_f_ (°C)	$\Delta$ T_f_ (°C)	T_f_ (°C)
26.0	0.78	74.7 $\pm$ 2.7	− 58.8 $\pm$ 4.1	15.9 $\pm$ 4.9
31.8	0.80	75.5 $\pm$ 1.6	− 55.6 $\pm$ 1.5	19.9 $\pm$ 2.2
50.9	0.85	72.2 $\pm$ 0.1	− 62.6 $\pm$ 1.8	9.6 $\pm$ 1.8
69.9	0.90	75.5 $\pm$ 3.2	− 28.8 $\pm$ 1.5	46.8 $\pm$ 3.6

**Figure 4:**
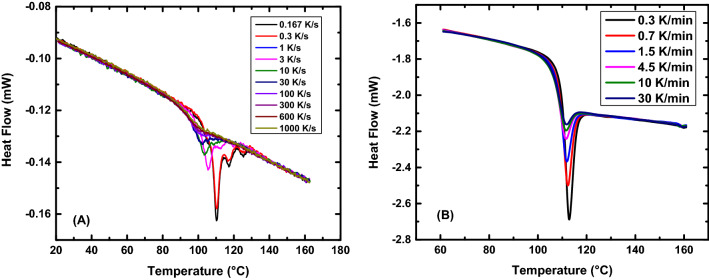
Heat flow curves at various cooling rates. (**A**) Heat flow scans for a 210 nm rejuvenated VPD film deposited at 0.85T_g_ and (**B**) heat flow scans for a 15.76 mg sample of virgin material.

A cooling rate dependence (CRD) of the fictive temperature of the rejuvenated VPD material was also carried out to determine the Vogel-Fultcher-Tammann (VFT) parameters, which includes a temperature at which the viscosity or relaxation time diverges to infinity. Figure 4 shows the heating scans for the rejuvenated VPD CYTOP and the virgin CYTOP.

**Figure 5:**
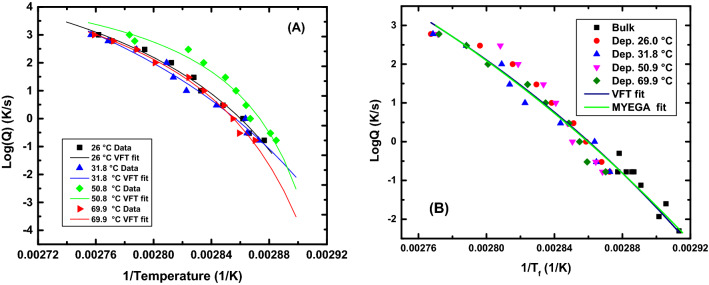
Cooling rate dependence of T_f_ for VPD and bulk CYTOP. (**A**) Cooling rate dependence of rejuvenated VPD CYTOP films along with their VFT fits. (**B**) All CRD data for the VPD CYTOP films and the virgin material CRD data along with the VFT and MYEGA fits on the data. Here, data for the deposition temperature of 50.9 °C have been shifted by + 3 °C and the virgin (bulk) material fictive temperatures has been shifted by -29 °C. VFT parameters for individual data sets are presented in Section 2 of the Supplemental Information 1.

From the VFT parameters the fragility (m) and the apparent activation energy (E_g_) were calculated. Figure 5 shows the inverse of the fictive temperatures for the VPD CYTOP as a function of cooling rate at different deposition rates along with their respective VFT fits. Figure 5 also shows the results for CRD for the virgin CYTOP. We also note that Badrinarayanan et al.^61^ compared T_g_ values measured on cooling with the limiting fictive temperature, T_f_′, measured on heating and found that the T_f_′ is systematically lower than T_g_, citing the breadth of the relaxation on cooling presumably as the cause. The CRD T_f_ data for the virgin material were shifted by − 29 °C, which is the difference between the T_g,bulk_ and the VPD T_f_ at nominal cooling rates of 10 K/min. From the intrinsic viscosity measurements. The results showed us that the material is still polymeric in nature, which allows us to assume that the shape of the VFT would be the same. Also, combining the FDSC and conventional DSC data provides a larger number of decades, in terms of cooling rate, which gives a better determination of the VFT parameters and the fragility^62^.

The CRD data set for the 50.8 °C deposition temperature was shifted by 3 °C to bring the fictive temperatures at the nominal cooling rate for samples of all deposition temperatures into alignment because of the variability in the chip response^60^. The VFT fit was applied to all the data, after alignment, and the parameters were calculated to be LogA = 16.6 $\pm$ 5.1, B = 1981 $\pm$ 1250 K, and ${T}_{\infty }$ = 297.2 $\pm$ 16.2 K. These results produced a fragility value of m = 116 and an apparent activation energy at T_g_ of E_g_ = 773 kJ/mol, which are similar values to those of high fragility polymers^63^. If we attempt to quantify the thermodynamic stability of the most stable glass created using the relationship shown in Eq. (12)^31^, and

12 $$\theta_{K} = \frac{{{\text{T}}_{{{\text{g}},{\text{ rejuv}}}} - {\text{T}}_{{{\text{f}},{\text{ stable}}}} }}{{{\text{T}}_{{{\text{g}},{\text{rejuv}}}} - {\text{T}}_{{\text{K}}} }}$$

equating ${\mathrm{T}}_{\mathrm{K}}={\mathrm{T}}_{\infty }$, we obtain a value for ${\theta }_{K}$ of the CYTOP 1.20 $\pm$ 0.38. A value of 1 for ${\theta }_{K}$ would nominally indicate that the molecules occupy lowest possible position in the energy landscape assuming that the ${\mathrm{T}}_{\mathrm{K}}$ or T_VFT_ correspond to the point of a thermodynamic glass transition, thus the present work suggests the possibility of creating a glass having a fictive temperature below the Kauzmann temperature. This further suggests that the fact that glass-formation in non-crystallizable materials such as the perfluoropolymer used here and that forms such an ultra-stable glass either form a different class of glass-forming materials than do crystallizing systems or that the Kauzmann paradox/paradigm/conundrum may not be very relevant to glass-formation itself. Table 2 summarizes an analysis of literature data for the values of ${\theta }_{K}$, for glasses stabilized in different ways. The present work is the only one we found for which ${\theta }_{K}$ > unity.

**Table 2:** Summary of some literature results for stability of glasses made by different paths.

Material	T_g_ (K)	T_f_ (K)	T_K_ (K)	${\uptheta }_{\mathrm{K}}$
20 Ma Amber^23^	409.4	365.8	338.4	0.61
PVAc^64^^ϕ^	313.2	302.5	250.0	0.17
TNB^31^	348.0	306.0	250.0	0.43
IMC^31^	315.0	286.0	240.0	0.39
Ethylbenzene^39^	116.6	103.3	101.0	0.85
Toluene^41^	117.5	106.0	100.0	0.66
AF 1600^50^	403.2	346.2	346.0	0.99
Ethylbenzene^65^	114.5	105.2	103.1	0.82
Polystyrene^45^	300.1	274.2	256.7	0.60
TNB^31^^ϕ^	348.0	324.5	250.0	0.24
IMC^31^^ϕ^	315.0	298.5	240.0	0.22
Polystyrene^66^^ϕ^	373.2	–	–	0.11
Pd_42.5_Cu_30_Ni_7.5_P_20_^67^^ϕ^	573.0	–	–	0.47
Au_49_Cu_26.9_Ag_5.5_Pd_2.3_Si_16.3_^68^^ϕ^	439.0	–	–	0.56
Ce_70_Al_10_Cu_20_^69^^ϕ^	347.2	307.2	290.8	0.71
CYTOP (current work)	348.3	285.8	297.2	1.20

The symbol ‘^ϕ’^ indicates data captured by conducting aging experiments. PVAc was annealed at 298 K for 2 months; TNB was annealed at 328 K for 15 days; IMC was annealed at 295 K for 210 days; Polystyrene was annealed at 358 K for 365 days; Au_49_Cu_26.9_Ag_5.5_Pd_2.3_Si_16.3_ was annealed at 373 K for 100,000 s; Pd_42.5_Cu_30_Ni_7.5_P_20_ was annealed at 540 K for 10,000 min; Ce_70_Al_10_Cu_20_ was annealed at room temperature for 6443 days. The ‘–‘ indicates value was not reported.

The results shown in this paper indicate that the stable CYTOP glass grown at a substrate temperature of 0.86T_g_ has a T_f_ of approximately 12.6 °C, which is 11.4 °C below the VFT divergence temperature ${\mathrm{T}}_{\infty }$ = 24 °C. The result of having a T_f_ value that is lower than the T_K_ questions underlying ideal glass transition theories. Is the Kauzmann paradox relevant to non-crystallizable glass formers? For example, Gibbs-DiMarzio^70,71^ theory hypothesizes that for non-crystallizable polymers, at a certain temperature below T_g_, the stiffening (shrinking) of the polymer chains ceases in order to leave the system with at least one configuration, i.e., the configurational entropy goes to zero. This temperature, called T_2_ has been related to an ideal glass transition and the Adam-Gibbs^72^ ansatz that relates the diverging dynamics to the decreasing entropy, viz., the VFT temperature and T_K_ (or T_2_) are related.

To our knowledge, this discovery is the first of its kind with regards to stable high MW polymers and questions classical theories that predict molecular behavior deep in the glassy region. Although we realize that there is uncertainty in our results, such a T_f_ reduction allows for a larger temperature range of experimentation to explore molecular dynamics far below T_g_. In addition, the possible observation that T_f_ < T_K_ suggests that the Kauzmann paradox may not be important in the understanding of glass-formation, at least in non-crystallizable materials such as that studied here. Consistent with this view are density data for vapor deposited ethylbenzene from Ishii et al.^73^ which McKenna^74^ analyzed and found that the fictive temperature is 8 K below the T_K._ This observation along with the present findings for the VPD CYTOP material pave an exploration avenue in the deep glassy state that is filled with challenges and interest.

Further investigations should be carried out to examine, e.g., density and dynamics of these ultra-stable glasses. AFM or ellipsometric thickness change dilatometry to explore thickness evolution of ultra-stable CYTOP films with temperature to obtain volumetric information to match with the enthalpy studies are such avenues of research. In addition, dynamic measurements such as the viscoelastic response provided from the TTU nanobubble inflation experiment^75,76^ are anticipated to provide the means to explore the dynamics of the ultrastable and extremely thin CYTOP films in the glassy regime, capturing relaxation behavior in the temperature range between T_f,VPD_ and T_g,rejuvenated_. It is also suggested that other polymers that might be more dielectrically active than the fluorinated materials used here and in prior VPD work would also provide a possibility to investigate the ultra-stable glasses in the deep glassy state.

The study of ultrastable glasses remains an area of research and the present work suggests there is a regime in the case of vacuum pyrolysis deposited polymers to create systems of higher stability than heretofore reported. In this work we used calorimetry measurements to investigate the T_f_ reduction. The origins behind why T_f_ of the stable VPD glasses below T_dep_ are not known and clearly require further exploration. We think that this shows that the vapor deposition is a powerful method of creating ultrastable polymer glasses, as originally suggested by McKenna and co-workers^50, 76^ using an amorphous fluoropolymer and subsequently confirmed by Raegen et al.^45^ for polystyrene and poly(methyl methacrylate).

## Summary and conclusions

We have created ultra-stable glassy CYTOP films using vapor pyrolysis deposition at varying substrate temperatures. Thickness of the stable films grown varied from 185 to 220 nm and the substrate temperature varied from 26 to 69.9 °C. FTIR spectroscopy indicated that both VPD films and the virgin material are of the same chemical composition and structure. Intrinsic viscosity measurements showed a MW decrease of approximately 50% but the material was still polymeric in nature. Heat flow scans of the stable VPD CYTOP films showed large enthalpy undershoots, one major signature of stable glasses. The stable CYTOP film deposited at a deposition temperature corresponding to T_dep_/T_g,Bulk_ = 0.86 exhibited a T_f_ reduction of 62.6 °C relative to the glass temperature, making T_f_ of the stable film lower than the VFT divergence temperature by 11.4 °C. The work provides a potential challenge to ideas related to the ideal glass transition that are based on the Gibbs-DiMarzio model and on the the Kauzmann paradox and suggests a route to making deep glassy state materials that can provide important new avenues of research and understanding.

## Supplementary Information

Supplementary Information.

## Data Availability

The datasets used and/or analyzed during the current study are available from the corresponding author (GBM) upon reasonable request.
