# Supplementary material for: Challenging the Kauzmann paradox using an ultra-stable perfluoropolymer glass with a fictive temperature below the dynamic VFT temperature
Source: Sci Rep. 2023 Mar 14;13:4224. doi: 10.1038/s41598-023-31074-0 (PMC10014873; doi:10.1038/s41598-023-31074-0)
Supplement: Supplementary file 1 — Supplementary Information. [file 41598_2023_31074_MOESM1_ESM.docx]

**Supplemental Information**

**Challenging the Kauzmann Paradox using an Ultra-Stable Perfluoropolymer Glass with a Fictive Temperature below the Dynamic VFT Temperature**

**Amer A. El Banna^1^ and Gregory B. McKenna*^1,2^**

**^1^Texas Tech University, Lubbock, TX, USA**

**^2^North Carolina State University, Raleigh, NC, USA**

**1 - Branching**

Another possibility that would give rise to a reduction in apparent molecular weight would be branching of the polymer structure as repolymerization occurs on the substrate surface. Such branching could also explain the reduction in the relative and specific viscosities. To investigate this possibility, we assume that branching is the main reason for the T_g_ reduction between the bulk CYTOP and the rejuvenated VPD films. We use a ratio of the intrinsic viscosities ($g^{'}$) of the VPD material and the virgin material to determine whether the VPD CYTOP is consists of either branched or linear chains. For branched polymers, the relationship that relates the ratio of radius of gyration ($R_{g}$) of the branched and linear structures to $g^{'}$ is shown in eqs. 1 and 2:

$g={g'}^{\varepsilon}$ (1)

$g=\frac{R_{g,Branch}}{R_{g,Linear}}, g^{'}=\frac{\left[ \eta\right]_{Branch}}{\left[ \eta\right]_{Linear}}=\frac{\left[ \eta\right]_{VPD}}{\left[ \eta\right]_{Virgin}}$ (2)

where $\varepsilon$ ranges from 0.5 – 1.5^1-3^.

Also, $g$ is related to the functionality ($f$) of branched polymers, assuming the branched structure is the monodisperse star shape polymer, by eq. 3^52^:

$g=\frac{3}{f}-\frac{2}{f^{2}}$ (3)

In this work, $g^{'}$ is 0.61 which in turn computes $f$ values in the range between 3 – 6.

We also use the Fox-Flory equation^4^ (Eq. 4) to estimate the reduction T_f_ for the VPD CYTOP

$T_{g}=T_{g}^{\infty}-\frac{B}{M_{n}}, where B=\frac{2\theta\rho N_{A}}{\alpha_{f}}$ (4)

Here $T_{g}^{\infty}$ is the $T_{g}$ of infinite MW material, 2$\theta$ is the excess free volume per linear molecule of the chain ends, $\rho$ is the density of the material (2.03 g/cm^3^), $N_{A}$ is Avogadro’s constant and $\alpha_{f}$ is the coefficient of expansion for free volume of the material. Applying eq. 14 for both the VPD and the virgin material then subtracting them from each other yields eq. 5:

$M_{n,VPD}=\frac{1}{\frac{\left( T_{g, Virgin}-T_{g, PVD} \right)}{B}+\frac{1}{M_{n,Virgin}}}$ (5)

Using $\alpha_{f}$ = 4 $\times$ 10^-4^ K^-1^ and $\theta$ ranging from 180 – 1150 Å^3^, calculated using the reported free volume hole radius for CYTOP (3.5 – 6.5 Å)^5^, computes an M_n_ value in the range between 29,047 – 92,657 g/mol, which is consistent with the intrinsic viscosity results.

Now, revisiting the idea that branching would be responsible for the reduction in T_g_, we us eq. 6 to analyze its validity.

$T_{g,bulk}-T_{g, VPD(star)}=\frac{\mathrm{fB}}{{2M}_{n,VPD}}=\frac{f}{2}\left( T_{g,bulk}-T_{g, VPD(linear)} \right)$ (6)

Using $f$ values calculated earlier ($f$ = 3 – 6), we find that the expected T_g_ reduction (left hand side of eq. 16) is in the range of 54.5 – 227.6 K, which is much greater than the value of 29 K measured in the present work. If we also use eq. 16 to predict the functionality $f$ for a 29 K T_g_ reduction, we find that $f$ values would range between 0.68 – 1.60, which indicates that the material is linear in structure.

**2 - VFT parameter values**

| $T_{\mathrm{Deposition}} (℃)$ | $\frac{T_{\mathrm{Deposition}} (K)}{T_{g Bulk - reported} (K)}$ | $A$ | $B (K)$ | $T_{0} (K)$ | *m* |
| --- | --- | --- | --- | --- | --- |
| $26.0$ | $0.78$ | $5.8\pm0.6$ | $66.8\pm15.0$ | $338.1\pm1.3$ | $7.2\pm1.7$ |
| $31.8$ | $0.80$ | $12.4\pm8.0$ | $451.6\pm630.2$ | $313.6\pm26.0$ | $18.3\pm29.6$ |
| $50.9$ | $0.85$ | $5.9\pm1.2$ | $60.2\pm28.0$ | $339.8\pm2.2$ | $7.1\pm3.2$ |
| $69.9$ | $0.90$ | $7.0\pm1.4$ | $110.3\pm49.4$ | $334.5\pm3.5$ | $9.9\pm4.7$ |
| $Bulk$ | $NA$ | $7.3\pm19.0$ | $181.5\pm768.0$ | $324.3\pm39.2$ | $10.6\pm47.8$ |

**References**

(1) Zimm, B. H., Stockmayer, W. H., The Dimensions of Chain Molecules Containing Branches and Rings. *J. Chem. Phys*., 17 (12), 1301−1314 (1949).

(2) Zimm, B. H. & Kilb, R. W. The Dynamics of Branched Polymer Molecules in Dilute Solution. J. *Polym. Sci*., 37 (131), 19−42 (1959).

(3) Graessley, W. W. & Mittelhauser, H. M., Intrinsic Viscosity of Polydisperse Branched Polymers. *J. Polym. Sci*., Part B: *Polym. Phys*., 5 (3), 431−454 (1967).

(4) McKenna, G.B. Glass Formation and Glassy Behavior. *Comprehensive Polymer Science*, Vol. 2: Polymer Properties, ed. by C. Booth and C. Price, 311-362 (1989).

(5) G. Dlubek, J. Pionteck, M. Sniegocka, E. M. Hassan, R. Krause-Rehberg, Temperature and Pressure Dependence of the Free Volume in the Perfluorinated Polymer Glass CYTOP: A Positron Lifetime and Pressure-Volume-Temperature Study. Journal of Polymer Science: Part B: Polymer Physics, Vol. 45, 2519–2534 (2007).
